# Two Type VI Secretion DNase Effectors are Utilized for Interbacterial Competition in the Fish Pathogen *Pseudomonas plecoglossicida*

**DOI:** 10.3389/fmicb.2022.869278

**Published:** 2022-04-06

**Authors:** Yanyan Li, Xiaojun Yan, Zhen Tao

**Affiliations:** School of Fisheries, Zhejiang Ocean University, Zhoushan, China

**Keywords:** *Pseudomonas plecoglossicida*, type VI secretion system, VgrG, DNase, antibacterial activity, interbacterial competition, T6SS

## Abstract

*Pseudomonas plecoglossicida* is a facultative fish pathogen that possesses three distinct type VI secretion systems (named T6SS-1, T6SS-2, and T6SS-3). Our previous work indicated that only T6SS-2 of *P. plecoglossicida* mediates interbacterial competition. However, the antibacterial T6SS effectors and their functions are unclear. Here, we reported two T6SS effectors that mediate antibacterial activity. We first identified four putative antibacterial effectors (denoted as Txe1, Txe2, Txe3, and Txe4) and their cognate immunity proteins encoded in *P. plecoglossicida* strain XSDHY-P by analyzing the regions downstream of three *vgrG* genes. We showed that the growth of *Escherichia coli* cells expressing Txe1, Txe2, and Txe4 was inhibited, and these three effectors exhibited nuclease activity *in vivo*. The interbacterial competition assays with single- or multi-effector deletion mutants as attackers revealed that Txe1 was the predominant T6SS toxin of *P. plecoglossicida* strain XSDHY-P mediating the interbacterial killing. This work contributes to our understanding of bacterial effectors involved in the interbacterial competition.

## Introduction

To survive and reproduce, bacteria need to compete with other microorganisms for nutrients and space. Many Gram-negative bacteria can utilize toxic proteins, known as “effectors” translocated by the type VI secretion system (T6SS) to eliminate their competitors (Basler et al., 2013; Cianfanelli et al., 2016; Hernandez et al., 2020). The T6SS is a contractile puncturing device with a needle-like structure, consisting of contractile sheath, an inner tube (composed of stacked rings of Hcp hexamers), and a transmembrane-baseplate complex (Mougous et al., 2006; Leiman et al., 2009). The Hcp tube is packed in the sheath. On top of the Hcp tube is a trimeric VgrG spike on which sits a cone-shaped proline–alanine–alanine–arginine repeat family (PAAR) domain-containing protein sharpening the tip (Cascales and Cambillau, 2012; Shneider et al., 2013). In response to unclear extracellular signals, the sheath contracts causing the needle structure to be expelled and penetrate the target membrane, thereby translocating effectors into neighboring bacterial or eukaryotic cells (Cascales and Cambillau, 2012; Jurėnas and Journet, 2021).

The T6SS needle structure carries effectors occurring in two modes. One class of effectors comprises covalent extensions of one of the components of the needle structure (Hcp, VgrG, or PAAR), and these are coined as “specialized” or “evolved” effectors. In contrast, “cargo” effectors non-covalently interact with Hcp, PAAR, or VgrG proteins. Often, adaptor or chaperone proteins are essential for stabilization and/or recruitment of effectors during assembly (Unterweger et al., 2017; Manera et al., 2022). Some of the T6SS adaptor proteins are those belonging to the DUF1795, DUF2169, and DUF4123 families. DUF1795 family proteins, termed Eag (effector-associated gene) adaptors, bind to the N-terminal half of PAAR-containing T6SS effectors, acting as a chaperone to stabilize the transmembrane domains of “specialized” effectors during the translocation across bacterial cells (Diniz et al., 2015; Whitney et al., 2015; Jurėnas et al., 2021). DUF2169 and DUF4123 family proteins seem to be less prevalent and were shown to assist the loading of “cargo” effectors onto the VgrG spike (Unterweger et al., 2015; Bondage et al., 2016; Pei et al., 2020).

Antimicrobial properties exist in many T6SS effectors, including those toxins with activities of nucleases, phospholipases, and peptidoglycan hydrolases (Durand et al., 2014). These effectors are toxic to essential cellular targets and cause either lysis or death of the attacked bacterial cells. Antibacterial effectors are often accompanied by cognate immunity proteins that confer self-protection (Russell et al., 2011) while preventing the bacteria from being damaged by sibling cells (MacIntyre et al., 2010). The antibacterial effector genes are often found immediately upstream of cognate immunity genes (Lewis et al., 2019).

Recently, we reported that a fish pathogenic *Pseudomonas plecoglossicida* strain carries three T6SS clusters (T6SS-1, T6SS-2, and T6SS-3), and each encodes a full set of core T6SS components (Tao et al., 2020). Our study specifically indicated that T6SS-2 participates in bacterial killing and is conserved across strains from either fish host or environmental sources. The T6SS-2 activity lends a competitive advantage to *P. plecoglossicida* over bacteria such as *Photobacterium damselae* subsp. *damselae* that is probably a competitor for host fish niches (Tao et al., 2020). It is unclear which effectors are deployed by *P. plecoglossicida* T6SS-2 to eliminate bacterial competitors from similar environments. This work aims to identify and characterize T6SS-2-dependent antibacterial effectors used by a fish pathogenic *P. plecoglossicida* strain XSDHY-P.

## Materials and Methods

### Bacterial Strains and Growth Conditions

Bacterial strains used are listed in Supplementary Table S1. *Pseudomonas plecoglossicida* strain XSDHY-P (Zhang et al., 2014) and its derivatives were routinely grown in Luria–Bertani (LB) broth or solid medium (supplemented with 1.5% agar) at 28°C. *Escherichia coli* strain DH5α was used for plasmid maintenance and amplification. *Escherichia coli* strain BL21 (DE3) was used in growth inhibition assays. *Escherichia coli* strain XL10 (purchased from Vazyme Biotech, Nanjing, China) was used as the prey strain in interbacterial competition assays. The *E. coli* strains were cultured at 37°C and were grown in/on LB broth or agar plates at 28°C. Media were supplemented with the following antibiotics as required: ampicillin at 100 μg/ml, chloramphenicol at 25 μg/ml, kanamycin at 50 μg/ml, and tetracycline at 15 μg/ml.

### Identification of Putative Antibacterial T6SS Effectors in *Pseudomonas plecoglossicida* XSDHY-P

The known antibacterial T6SS effectors are often encoded in the vicinity of or fused to *vgrG* genes (Hachani et al., 2014; Bernal et al., 2017; Wettstadt et al., 2019; Wood et al., 2019). Consequently, to identify putative effector and immunity proteins, we first searched for all putative *vgrG* genes encoded in *P. plecoglossicida* XSDHY-P. To this end, we used amino acid sequences of previously identified VgrG-1 (DVB73_RS06775), VgrG-2 (DVB73_RS03075), and VgrG-3 (DVB73_RS25130) to perform TBLASTN (Altschul et al., 1997) queries against a database of translated protein sequences belonging to the single strain XSDHY-P at www.pseudomonas.com with default settings (Winsor et al., 2016). Open-reading frames (ORFs) presented in *vgrG* gene neighborhoods were manually inspected using BLASTp searches against the NCBI “nr” database and analyzed for conserved domains using the NCBI Conserved Domain Database (Marchler-Bauer et al., 2017) tool for conserved effector domains. The SecreT6 webtool for T6SS-related protein prediction was used to search putative effector and immunity proteins against the database using an *E*-value threshold of e-4 (Li et al., 2015). Only those putative effectors encoded with downstream immunity proteins were included for further analysis. A putative immunity protein was identified using the following criteria: (a) the protein was encoded downstream (within 50 bp) of the cognate effector gene and (b) the length of the protein was at least 50 amino acids and was smaller than its cognate effector. PSORTb (v3.0.2) was used to predict subcellular localization of putative immunity proteins (Yu et al., 2010), thereby inferring the location of their cognate effectors, since immunity proteins are required in the correct cellular compartment of effector-producing cells to neutralize effector toxicity for self-protection. TMHMM (v.2.0) was used to predict transmembrane domains (Krogh et al., 2001), and SignalP 5.0 was used to predict signal peptides (Almagro Armenteros et al., 2019). BLASTp (Altschul et al., 1997) was used to calculate the identities between effector proteins. The deep-learning approach, AlphaFold2, was used to predict protein tertiary structures (Jumper et al., 2021). ESPript was used to visualize the sequence alignment (Robert and Gouet, 2014).

### Phylogenetic Analysis of Proteins

The amino acid sequences of analyzed proteins were multi-aligned with ClustalOmega (Sievers et al., 2011). The maximum likelihood (ML) phylogenetic tree was constructed using IQ-TREE (Trifinopoulos et al., 2016) under the LG + F + I + Gamma model, with 1,000 ultrafast bootstrap replicates (Minh et al., 2013). The phylogenetic tree was visualized in iTOL (Letunic and Bork, 2016).

### Plasmid Construction for Arabinose-Inducible Expression

Plasmids used are listed in Supplementary Table S1. To analyze the effector/immunity activity in *E. coli*, coding regions for putative effectors with or without cognate immunity genes were amplified from *P. plecoglossicida* XSDHY-P genomic DNA using Phanta® Max Super-Fidelity DNA Polymerase (Vazyme Biotech, China). The amplified fragments were cloned into the multiple cloning site (MCS) of the L-arabinose inducible vector, pBAD33.1 (Addgne#36267) either by Gibson assembly (Gibson et al., 2009) or by restriction cloning. For truncation analysis of effectors, the C-terminal domain of each effector coding sequence was inserted into pBAD33.1. Plasmid constructs were transformed into *E. coli* BL21(DE3) and confirmed by DNA sequencing. All primers used are listed in Supplementary Table S2.

### Generation of Deletion Mutants of *Pseudomonas plecoglossicida* XSDHY-P

In-frame deletion mutants of *P. plecoglossicida* were generated by two-step allelic exchange based on the suicide vector pK18*mobSacB* (Kvitko et al., 2009) as previously described (Tao et al., 2020). Briefly, 500–1,000 bp upstream and downstream of the gene to be deleted were amplified and then fused by overlapping PCR. The fused fragments were cloned into the suicide vector to construct the mutation plasmids (Supplementary Table S1). Mutation plasmids were transferred to *P. plecoglossicida* by conjugation. All mutants were verified by PCR. Mutants with multiple deletions were generated stepwisely. For multiple-deletion mutants, target genes were knocked out in a stepwise manner. The double-mutant Δ*txe1*Δ*txe4* was generated by deleting *txe4* on the Δ*txe1* genetic background. To obtain the triple-mutant Δ*txe1*Δ*txe2*Δ*txe4*, *txe2* was knocked out from the Δ*txe1*Δ*txe4* mutant.

### Bacterial Toxicity Assay

A bacterial toxicity assay was performed as previously described with several modifications (Jana et al., 2019). To test the toxicity of putative antibacterial effectors, *E. coli* BL21(DE3) with recombinant plasmids or an empty pBAD33.1 vector was streaked on LB agar containing either 0.2% glucose (repressing conditions) or 0.2% L-arabinose (inducing conditions) and chloramphenicol. The agar plates were incubated at 37°C. Images were acquired after 24 h.

To verify the protection conferred by immunity proteins, pBAD33.1 carrying both predicted effector and downstream immunity genes was transformed into *E. coli* BL21(DE3). Overnight cultures of *E. coli* cells were diluted 1:100 into fresh LB medium containing chloramphenicol and grown for 3 h at 37°C, 150 rpm. Each culture was normalized to an OD of 1.0 and diluted 100-fold in fresh LB with same antibiotics and divided into three sterile flasks as biological replicates. The initial OD_600_ was measured, and the flasks were incubated at 37°C, 150 rpm. After a 1.5-h incubation, *E. coli* cells were induced by adding 20% L-arabinose to a final concentration of 0.2% (wt/vol). The cultures continued to be grown under same conditions up to 9 h, and the OD_600_ was measured each hour since 30 min post-induction. A growth curve was plotted as the optical density (OD_600_) vs. the culture time. In addition, 10-fold dilutions were made from one replicate of each culture after incubation. Five-microliter aliquots of the dilutions was dropped onto LB agar to estimate the number of viable cells.

### Plasmid DNA Degradation Analysis

The plasmid DNA degradation analysis in *E. coli* cells was carried out as described previously (Ma et al., 2014). In brief, *E. coli* BL21(DE3) with recombinant or empty pBAD 33.1 have grown in LB containing chloramphenicol to an OD_600_ of 0.6–0.8. The expression of putative effector alone (or with cognate immunity) was then induced by 0.2% L-arabinose for 3 h. Equivalents of 1 ml of cells at OD_600_ = 1.0 from each culture were pelleted for plasmid extraction. Plasmid DNA was extracted using an E.Z.N.A. Plasmid DNA Mini Kit (Omega Bio-Tek, Inc., GA, United States). Plasmid DNA samples were analyzed by gel electrophoresis on a 1% agarose gel containing 1× GelRed (Biotium, Hayward, CA, United States) and run at 10 V/cm for 30 min. DNA was visualized under UV light transillumination using a Gel Doc system (BioRad, La Jolla, CA, United States).

### Interbacterial Competition Assay

Interbacterial competition assays were performed as previously described (Tao et al., 2020). In brief, wild-type *P. plecoglossicida* XSDHY-P and indicated mutants (sensitive to tetracycline) were used as attackers, and *E. coli* XL10 (resistant to tetracycline), which is susceptible to *P. plecoglossicida* T6SS-2-dependent killing (Tao et al., 2020), was used as the prey. Overnight cultures of bacterial strains were subcultured (1:50) in fresh LB for 3 h. The subcultures were adjusted to an OD_600_ of 0.5 in PBS and mixed at a 10:1 ratio of attacker to prey. Mixtures of 25 μl were spotted in triplicate onto 0.45-μm filter membranes that were placed on LB agar. The plates were incubated for 4 h at 30°C. Bacterial cells were dislodged from the membranes into 1 ml LB in 15-ml tubes by vortexing. The prey cells were enumerated by counting tetracycline-resistant colony-forming units (CFUs). The fold change in prey CFUs was calculated by dividing CFUs of the input (at 0 h) by the output (at 4 h). The prey growth index (PGI) was defined as log2 fold change in prey CFUs, which is a measure of how efficient an attacker strain is at prey-strain killing. A higher PGI indicates a lower ability of the attacker to kill the prey strain. The interbacterial competition assay was performed in triplicate.

### Statistical Analysis

Statistical analyses and figure production were performed using GraphPad Prism (version 8). One-way ANOVA, followed by Fisher’s least significant difference (LSD) test, was used to compare the PGI values between each effector mutant strain and wild-type strain or T6SS-2 inactivate mutant in the interbacterial competition assay. The differences were considered significant at *p* < 0.05.

## Results

### XSDHY-P Encodes Two Orphan *vgrG* Genes

**Figure 1:**
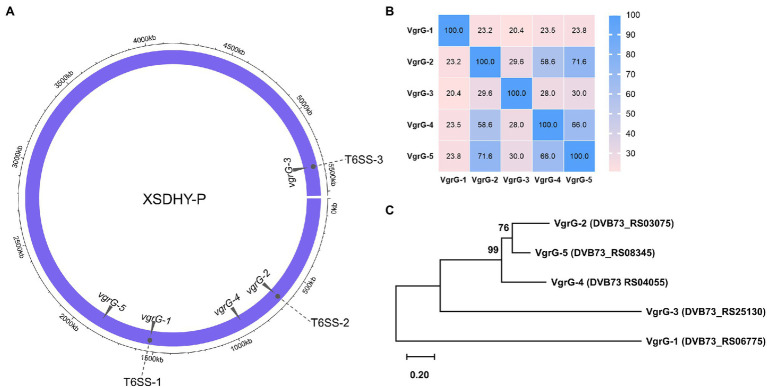
Distribution of the T6SS-1, T6SS-2, and T6SS-3 clusters, and the *vgrG* genes in the *Pseudomonas plecoglossicida* strain XSDHY-P **(A)**. Heatmap showing identity comparison matrix of sequence alignment of VgrG proteins **(B)**. A color key indicates the correspondence between pairwise identities and the colors displayed in the matrix. Phylogeny of VgrG proteins **(C)**. Maximum likelihood tree showing the phylogenetic relationship of VgrG proteins in *P. plecoglossicida* XSDHY-P strain. Bootstrap *n* = 1,000 constructed based on ClustalOmega alignment of VgrG amino acid sequences.

T6SS effectors and cognate immunity proteins are frequently found to be genetically linked to *vgrG* genes (Hachani et al., 2011, 2014; Durand et al., 2014; Bondage et al., 2016; Ma et al., 2018; Lopez et al., 2020). We did TBLASTN searches using three known VgrGs as queries to identify all *vgrG* genes in strain XSDHY-P. We found two additional *vgrG* genes that were located remotely from three T6SS loci (Figure 1A). These two newly identified *vgrG* genes were arbitrarily named as *vgrG-4* (DVB73_RS04055) and *vgrG-5* (DVB73_RS08345). A pairwise analysis of amino acid identity showed that five VgrG proteins encoded by XSDHY-P shared 20.4%–71.6% identity; VgrG-5 (71.6%) had the highest sequence identity with VgrG-2, followed by VgrG-4 (58.6%; Figure 1B; Supplementary Figure S1). ML phylogenetic analysis of VgrGs from XSDHY-P showed that VgrG-5 and VgrG-2 were sister groups, together forming a cluster with VgrG-4, and they were separated from VgrG-1 or VgrG-3 (Figure 1C).

### Four Putative Antibacterial Effector–Immunity Pairs Were Identified in *vgrG* Gene Clusters

**Figure 2:**
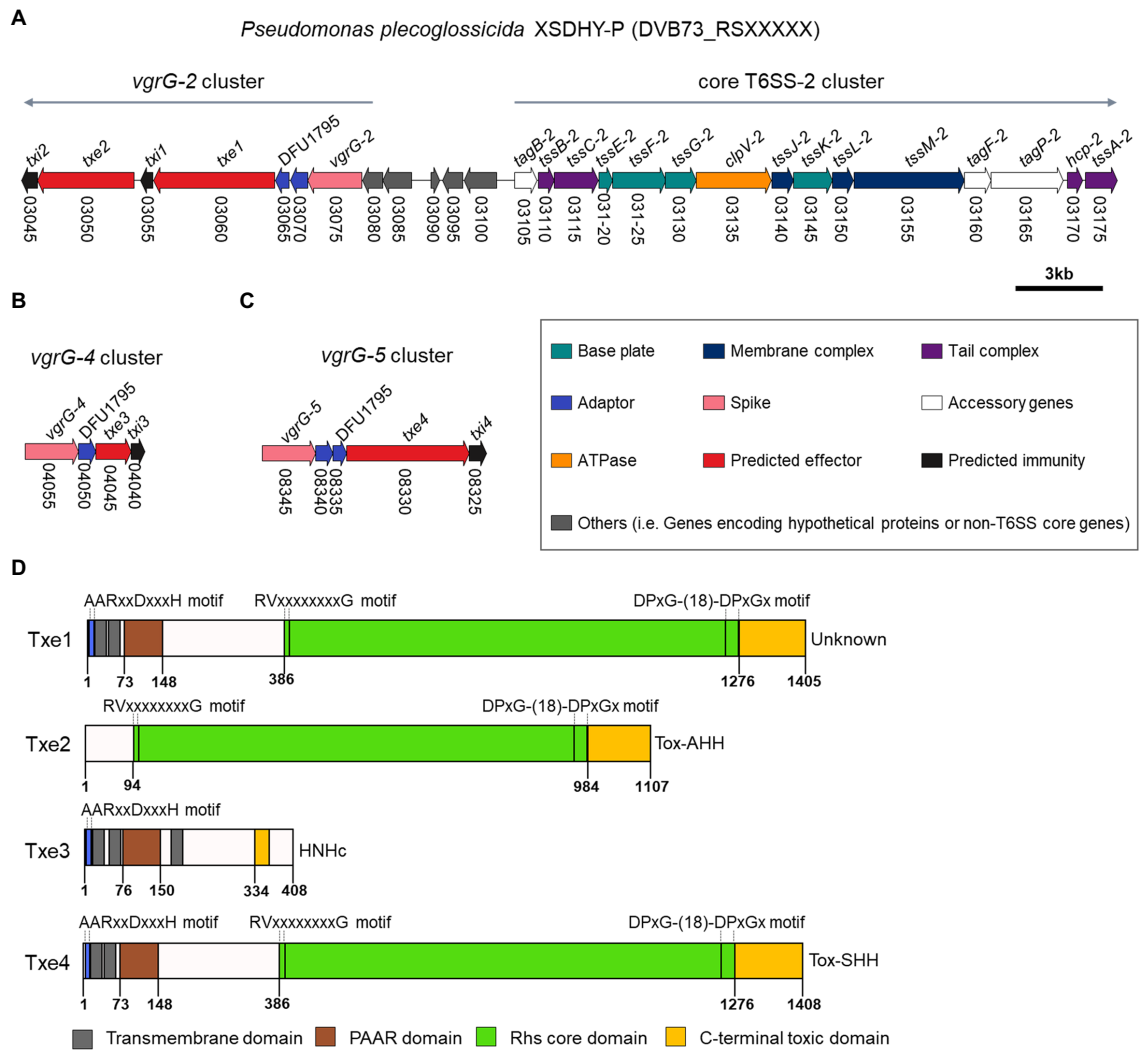
Genomic organization of T6SS-2 main cluster and *vgrG-2* cluster **(A)**, *vgrG-4* cluster **(B)**, and *vgrG-5* cluster **(C)** from *P. plecoglossicida* XSDHY-P. Genes are represented as arrows and the direction represents the direction of transcription. Genes are color-coded to highlight their predicted functions as described in the legend; colors specific to each gene are used to indicate genes that encode similar proteins in other clusters. Gene nomenclature is indicated at the top, and locus numbers are shown at the bottom. Predicted domain composition of four putative antibacterial T6SS effectors identified from *P. plecoglossicida* XSDHY-P **(D)**. Prediction was determined using the NCBI Conserved Domain Database tool (Marchler-Bauer et al., 2017). Schematic diagrams show the linear organization of relevant domains of putative effectors Txe1, Txe2, Txe3, and Txe4. The domains shown are: prePAAR motif (blue); transmembrane helices (dark grey); PAAR domain (brown); Rhs core domain (green); and C-terminal toxic domain (orange) as described in the legend. The lengths of the effector proteins and different domains are drawn to scale.

We inspected ORFs within *vgrG-2*, *vgrG-4*, and *vgrG-5* clusters for the presence of potential antibacterial E/I pairs. The *vgrG-1* and *vgrG-3* clusters were not analyzed, since T6SS-1 and T6SS-3 are not involved in antibacterial activities (Tao et al., 2020). A closer inspection of genes from relevant *vgrG* clusters allowed us to identify two putative E/I pairs within *vgrG-2* cluster (Figure 2A) and one E/I pair within *vgrG-4* and *vgrG-5* clusters each (Figures 2B,C) according to the aforementioned criteria. These putative Type six XSDHY-P effector and immunity proteins were named Txe1 to 4 and Txi1 to 4, respectively. Notably, gene *txe2*, DVB73_RS03050 was previously annotated as a pseudogene, yet manual inspection of the ORF revealed an intact gene (positions 14,787–11,465 in reference sequence NZ_CP031146.1). Between *vgrG* and effector genes, one or two EagR adaptor (DUF1795 domain-containing protein) genes were identified. There was a striking similarity between *vgrG-2* and *vgrG-5* clusters in terms of genetic organization, as well as amino acid sequences of protein counterparts encoded between the two clusters (Figure 1B; Supplementary Figure S1). For example, EagR adaptor proteins encoded between *vgrG-2* and *vgrG-5* clusters were almost identical that EgaR-2a and EgaR-5a shared 96% amino acid identity, and EagR-2b and EagR-5b were identical (Supplementary Figure S2). In contrast, the homology of EagR-2a, EagR-2b, EagR-5a, and EagR-5b to EagR-4 from *vgrG-4* cluster was low (22%–27% sequence identity). Considering the interaction between VgrG and Hcp proteins during T6SS assembly (Silverman et al., 2012) and the role of EagR in effector loading (Jurėnas and Journet, 2021), these data suggest that *vgrG-2-* and *vgrG-5-*associated effectors are likely linked to the same T6SS apparatus (i.e., T6SS-2) in strain XSDHYP.

The immunity protein is required in the correct cellular compartment for protection against self- and/or sibling damage by its cognate effector (Lewis et al., 2019). Consequently, to infer the subcellular target locations of the putative effectors, we predicted the subcellular locations of potential immunity proteins, Txi1, Txi2, Txi3, and Txi4 using PSORTb and SOSUI GramN tools. All four putative immunity proteins were predicted to be cytoplasmic by at least one tool (Supplementary Table S3). Signal peptide analysis using the SignalP 5.0 program revealed no signal peptides present in any of immunity proteins (Supplementary Table S3). Moreover, none of the immunity protein contained transmembrane domains (Supplementary Table S3). These results suggest that the effectors Txe1, Txe2, Txe3, and Txe4 are likely to be toxic to bacterial cells in their cytoplasm.

### Txe1, Txe2, and Txe4 are DNase Toxins That Belong to Different Nuclease Families

Our analyses using the SecReT6 webtool (Li et al., 2015) revealed that Tke2 (an Rhs effector with an N-terminal PAAR domain and C-terminal HNH nuclease domain) from *P. putida* KT2440 (Bernal et al., 2017) was the best hit for Txe1, Txe2, and Txe4 with identity ranging from 87.4% to 90.8%. A sequence alignment analysis revealed that the four effectors harbored distinct CT domains (Supplementary Figure S3). To further predict the putative functions, we used sequence analysis to determine the domain structures of the four putative effectors. The domain architectures of each effector are shown in Figure 2D and Supplementary Table S3. Txe1 contains an N-terminal (NT) PAAR domain, a canonical Rhs (after “Rearrangement hot spots”) core domain [delimited by specific motifs matched to previously identified RVxxxxxxxxG and DPxG-(18)-DPxGx peptide motifs; Jackson et al., 2009; Hachani et al., 2014], and a C-terminal (CT) extension of 130 amino acids. The CT extension of Txe1 showed no similarity to any known domains in the CDD database. Txe2 contains a conserved Rhs core domain and a Tox-AHH toxin (pfam14412; HNH/ENDO VII nuclease superfamily). Txe3 contains an NT PAAR and a putative HNHc endonuclease (pfam01844). Txe4 has a similar domain architecture with Txe1, carrying a Tox-SHH toxin (pfam15652; HNH/Endo VII superfamily toxin with an SHH signature) at its C-terminus. In addition, Txe1, Txe3, and Txe4 encode prePAAR motif and TMDs (Figure 2D; Supplementary Figure S4). In addition, Txe1, Txe3, and Txe4 each contain a prePAAR motif and TMDs (Figure 2D; Supplementary Figure S4). It has been reported that prePAAR motifs are found in TMD-containing effectors, directing recruitment of cognate Eag proteins to facilitate folding and maintenance of the hydrophobic helices (Ahmad et al., 2020). According to the different number of TMDs, the effectors containing TMDs were proposed to be classified as Class I and Class II effectors (Ahmad et al., 2020). This proposed classification scheme assigns Txe1 and Txe4 to Class I and Txe3 to Class II. In addition, the potential toxic CT domains of Txe1, Txe3, and Txe4 are fused to each NT PAAR domain, so these three toxins are classified as “specialized” effectors (Durand et al., 2014).

**Figure 3:**
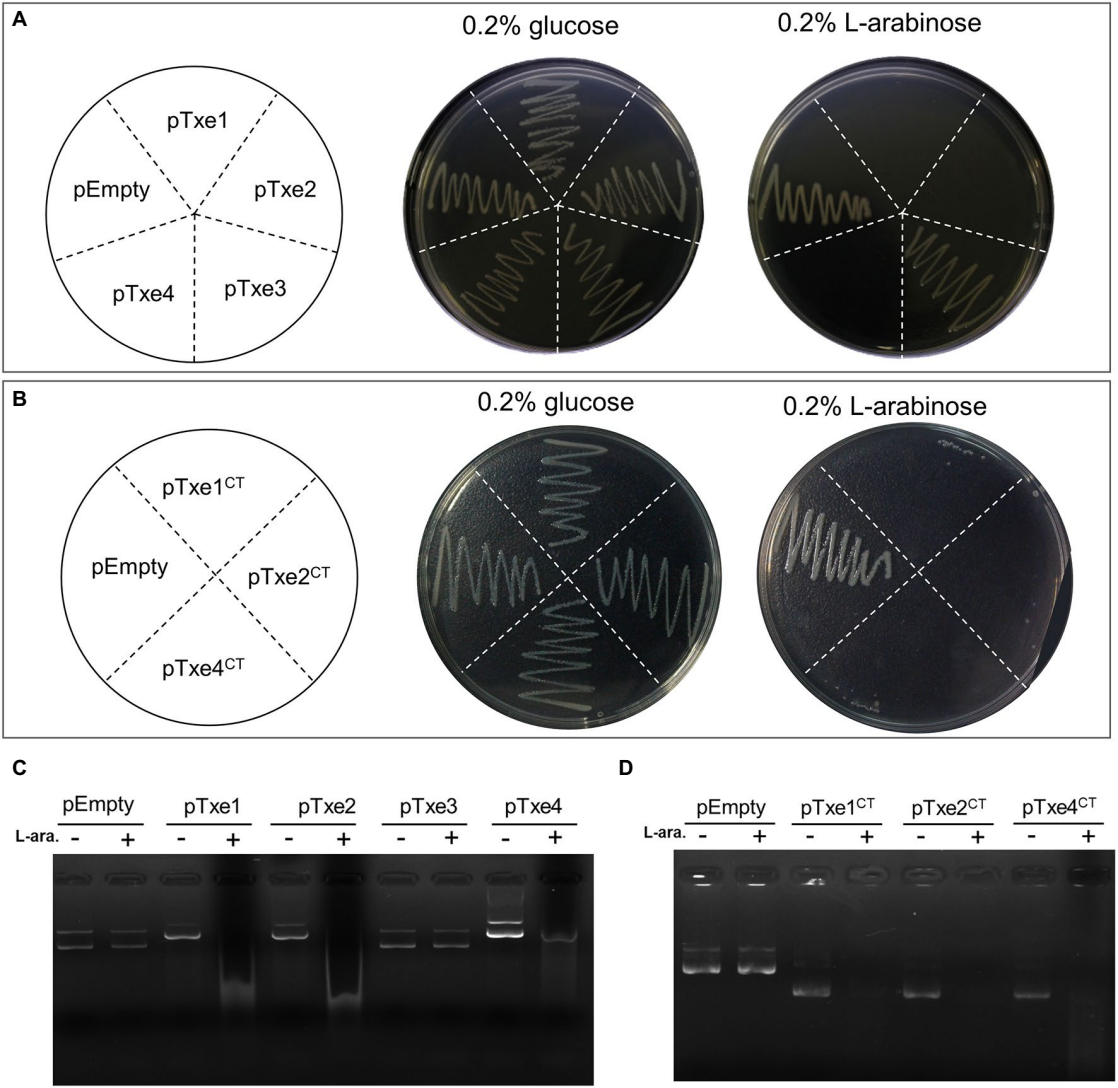
Txe1, Txe2, and Txe4 are three DNase toxins. Txe1, Txe2, or Txe4 were expressed on arabinose-inducible expression plasmid pBAD33.1 vector in *Escherichia coli* BL21(DE3), and the growth of *E. coli* was tested by streaking on LB agar containing 0.2% L-arabinose (induction) and/or 0.2% glucose (repression). The *E. coli* toxicity was not observed for expressed Txe3 **(A)**. Toxicity of CT domains of Txe1 (residues 1,276–1,405), Txe2 (residues 984–1,107), and Txe4 (residues 1,276–1,408) expressed in *E. coli* BL21(DE3) from arabinose-inducible expression plasmids **(B)**. Induced expression of full-length **(C)** or C-terminal extension **(D)** of Txe1, Txe2, or Txe4 for 3 h resulted in plasmid DNA degradation *in vivo*. Degradation was not observed for expressed Txe3. + And − symbols indicate addition or not of L-arabinose, respectively. The pBAD33.1 was transformed into *E. coli* BL21(DE3) to act as an empty vector control (pEmpty) for all assays. Representative data from three independent experiments are shown.

To validate the antibacterial activity of putative T6SS effectors, we cloned each Txe protein individually into the expression plasmid pBAD33.1 under the control of a P_BAD_ promoter (induced by L-arabinose and repressed by D-glucose) in *E. coli* BL21 (DE3). *Escherichia coli* cells were induced to express the four Txe proteins only for cytoplasmic localization, because the potential immunity proteins were predicted to be cytoplasmic. The results showed that induced expression of *txe1*, *txe2*, and *txe4* within the cytoplasm of *E. coli* was detrimental (Figure 3A), confirming that Txe1, Txe2, and Txe4 are cytoplasmic toxins. Unexpectedly, the expression of *txe3* did not arrest the growth of *E. coli*, though an HNHc endonuclease motif was predicted in Txe3. Truncation analysis demonstrated that heterologous expression of only the CT regions of Txe1 (residues 1,276–1,405), Txe2 (residues 984–1,107), and Txe4 (residues 1,276–1,408) was adequate to produce the observed toxicity (Figure 3B).

To assess the nuclease activity of the predicted effectors, we examined the degradation of the plasmid DNA extracted from *E. coli* strains after arabinose induction for 2 h. Expression of either *txe1*, *txe2*, or *txe4* for 3 h resulted in the degradation of plasmid DNA *in vivo* (Figure 3C) as well for their respective CT domains (Figure 3D). Consistent with the results of the toxicity assay, expression of *txe3* did not cause plasmid DNA degradation (Figure 3C). These results confirmed that Txe2 and Txe4 are DNase toxins as predicted from sequence analysis and demonstrated that Txe1 also has nuclease activity.

**Figure 4:**
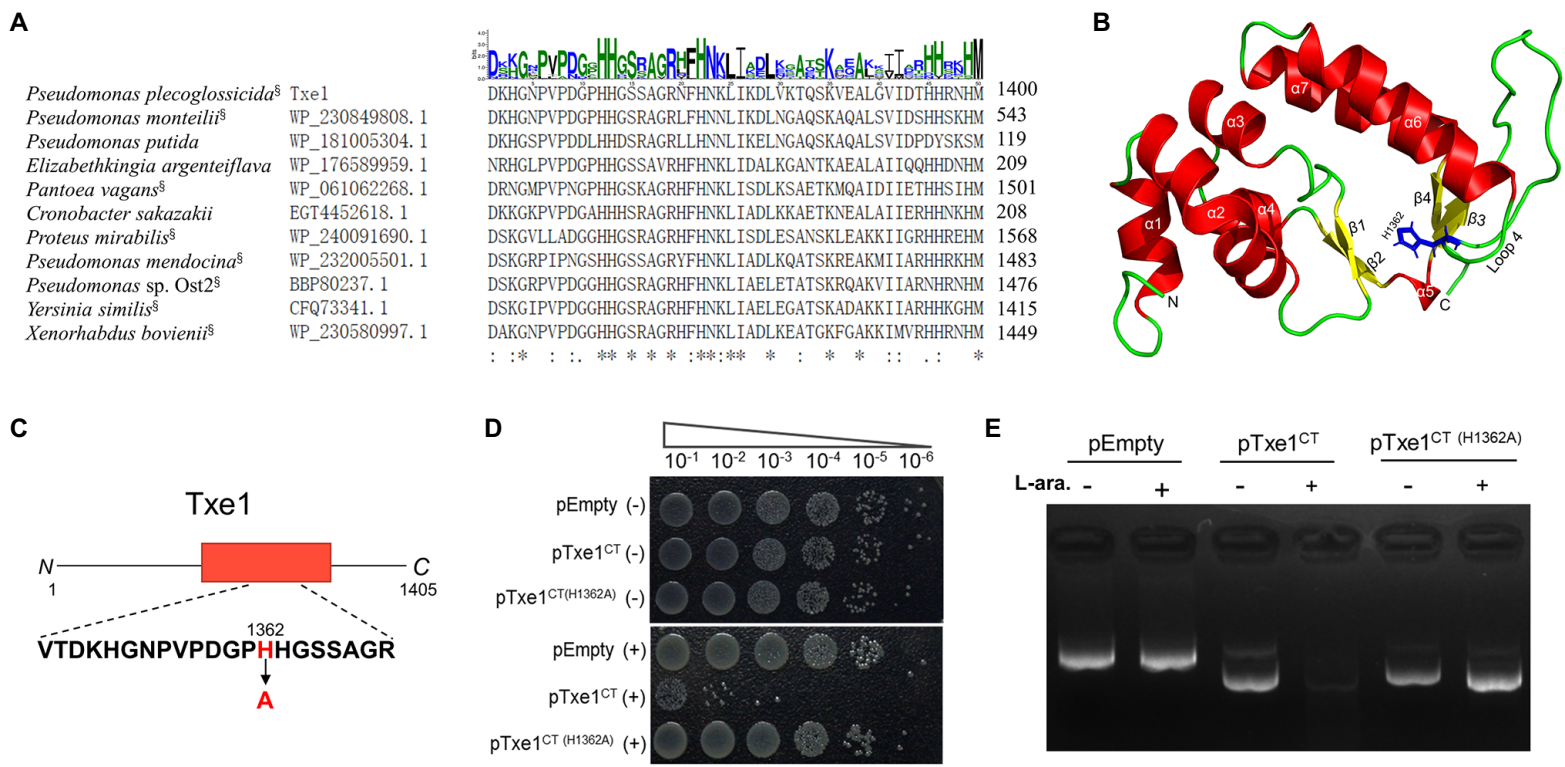
Mutation of H1362 abolishes toxicity of Txe1. Partial sequence alignment of Txe1 CT region with representative hits of BLASTp analysis **(A)**. In each case, the species name and accession number are on the left, and the numbering for the last residue is on the right. CT domains that are linked to N terminal Rhs domain are indicted with a superscript “§” behind the species name. On the top is the sequence logo representation of the aligned region of 50 residues (this region contains all the identical histidine residues in the protein sequence alignment of Txe1 CT domain with the 100 best BlastP hits.). Consensus sequences were plotted with WebLogo web-based tool (http://weblogo.threeplusone.com/create.cgi). Height of letter indicates degree of conservation. Residues identical in all species are indicated by “*” on the bottom. AlphaFold2-generated tertiary structure models for the CT domain of Txe1 **(B)**. Txe1 CT domain is colored by secondary structure: helices (red), sheets (yellow), and loop (green). A tertiary structure similar to the His-Me finger from caspase-activated DNase (PDB ID: 1v0d) is formed by sheets β3 and β4 and helices α6 and α7. Schematic representation indicating the mutation site of Txe1 **(C)**. Mutation of histidine 1,362 to alanine (H1362A) in Txe1 abolishes toxicity **(D)**. Growth of *E. coli* BL21(DE3) carrying empty pBAD33.1 (pEmpty) or plasmids directing the expression of the Txe1 CT domain or mutant on LB agar **(E)**. + And − symbols indicate addition or not of 0.2% L-arabinose, respectively. The experiment was repeated twice with similar results, and the results from one representative experiment are shown.

By using a query of Txe1 CT domain sequence, the BLASTp search revealed that homologs of this CT nuclease domain were also carried by some Rhs-repeats containing proteins from other bacterial species (data not shown). Partial sequence alignment of CT region of Txe1 with representative BLASTp hits showed that these proteins have a conserved dipeptide HH motif (H1362 and H1363; Figure 4A). These two conserved histidine residues are part of the loop (loop 4) connecting helix α6 and sheet β3 in a Txe1-CT domain structure model predicted by AlphaFold2 (Figure 4B). In this predicted structure model, sheets β3 and β4 and helices α6 and α7 together form the tertiary structure similar to the His-Me finger from caspase-activated DNase (PDB ID: 1v0d; Jablonska et al., 2017). Probably, the conserved dual histidine motif plays an indispensable role in the enzyme activity of Txe1. Here, we targeted H1362 and probed if it is import for the nuclease activity of Txe1. The histidine 1,362 was changed to alanine by site-directed mutagenesis (Figure 4C). As a result, the substitution of H1362 (H1362A) abolished the nuclease activity of Txe1 CT domain (Figure 4D) and its toxicity to host *E. coli* cells (Figure 4E). Hence, the residue H1362 is crucial for the DNase activity of Txe1.

### Txi1, Txi2, and Txi4 Antagonize the Toxicity of Txe1, Txe2, and Txe4, Respectively

**Figure 5:**
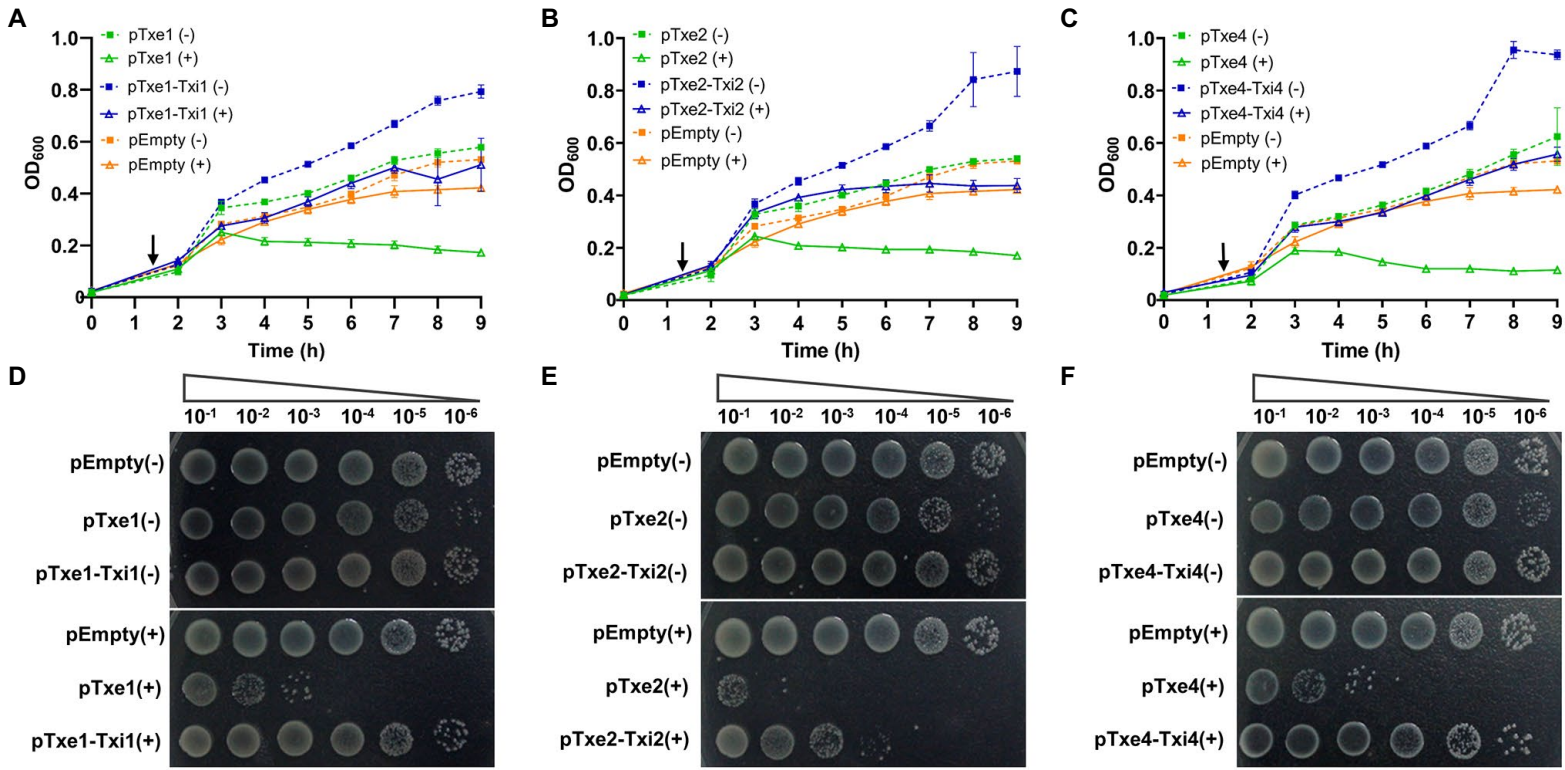
Txe1-Txei1, Txe2-Txei2, and Txe4-Txei4 are three effector–immunity pairs. Growth curves of *E. coli* BL21(DE3) harboring indicated plasmids were obtained by measuring the OD_600_ at 1-h intervals **(A–C)**. Data are presented as the mean ± SD of three independent experiments. Levels of survival of cells were compared by 10-fold serial dilution on LB medium containing arabinose or not **(D–F)**. The arrow indicates the time of induction with 0.2% L-arabinose, and (+) and (−) symbols indicate addition or not of L-arabinose, respectively. The experiment was repeated twice with similar results, and the results from a representative experiment are shown.

Antibacterial T6SS toxins are usually encoded along with a cognate immunity gene (Dong et al., 2013). To examine whether those proteins (i.e., Txi1, Txi2, and Txi4) encoded immediately downstream of *txe1*, *txe2*, and *txe4* are immunity proteins, we also cloned DNA fragments encoding each predicted E/I pair into the vector pBAD33.1. When induced with L-arabinose, Txe1, Txe2, and Txe4 were expressed in *E. coli* cytoplasm and exerted a bacteriostatic effect as measured by OD_600_ (Figures 5A–C). The growth inhibition of each effector was strikingly neutralized by the co-expression of the predicted immunity genes (i.e., *txi1*, *txi2*, and *txi4*; Figures 5A–C). Results of serial dilution spotting at the end of the experiment showed that viable cells of *E. coli* strains that co-expressed effector and immunity genes were 100–1,000 times higher than those that only expressed effector genes (Figures 5D–F). Moreover, the plasmid degradation analysis showed that co-production of Txi1, Txi2, and Txi4 partially suppressed the nuclease activity of cognate effectors (Supplementary Figure S5; Figure 3C). These results suggest that Txi1, Txi2, and Txi4 function as immunity proteins for Txe1, Txe2, and Txe4, respectively.

### Txe1 and Txe4 Mediate Interbacterial Competition in Strain XSDHY-P

**Figure 6:**
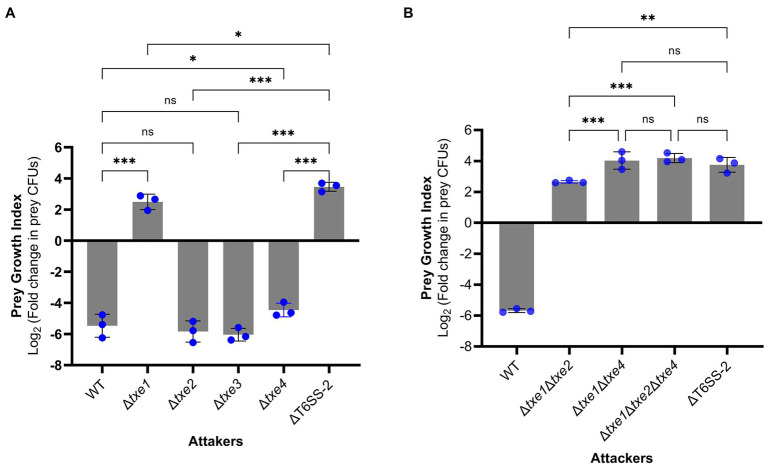
Interbacterial competition assay with *P. plecoglossicida* wild-type (WT) and deletion mutants as attackers. The prey growth index (PGI) was calculated from the prey strain *E. coli* XL10 when cocultured with *P. plecoglossicida* T6SS effector mutants or wild-type strain for 4 h. The PGI was defined as log2 fold change in prey CFUs (ratio of 4–0 h). A higher PGI indicates a lower ability of the attacker strain to kill the prey strain. PGI of single-effector deletion mutants vs. WT strain and ΔT6SS-2 **(A)**. PGI of a triple-effector deletion mutant vs. WT strain and ΔT6SS-2 **(B)**. Bars show mean ± SD of three biological replicates, with individual data points superimposed. Each data point represents the mean value of three technical replicates. The experiment was repeated twice with similar results, and the results from a representative experiment are shown. Comparison of PGI values between groups was performed using a one-way ANOVA and Fisher’s LSD test. ns, not significant (*p* > 0.05, ^*^p ≤ 0.05, ^**^p < 0.001, ^***^p < 0.001, and Student’s *t*-test).

To probe the role of four Txe effectors in XSDHY-P T6SS-2-related antibacterial activity, we generated a series of single- or multiple-effector deletion mutants. For all these mutants, only the indicated effector gene(s) were deleted, and the cognate immunity gene(s) were kept in the genome. We first assessed the contribution of each individual effector in interspecies competition. To do so, we performed interbacterial competition assays between Δ*txe1*, Δ*txe2*, Δ*txe3*, or Δ*txe4* (attacker strains) and *E. coli* strain XL10 (prey strain carrying tetracycline resistance). To measure the contribution of given effector(s) to T6SS killing, we determined the PGI, which reflects the survival of prey in co-culture with attacked strains on solid media. A high PGI indicates a higher survival rate for prey; a positive PGI value means a greater number of viable prey cells than baseline hour zero. Compared with the WT strain, the PGI was significantly higher in mutants deletion of *txe1* alone or in combination with *txe4* (*p* < 0.05), indicating that these two effectors contribute to the bacterial killing activity. Interestingly, no significant difference in PGI was observed between the WT strain and Δ*txe2* or Δ*txe3* (Figure 6A), although Txe2 encodes the toxic CT domain. Among all the single-deletion mutants, it was only when Δ*txe1* was used as the attacker that the PGI value was positive, indicating that the deletion of *txe1* attenuated T6SS killing the most (Figure 6A). The PGIs of Δ*txe1* and Δ*txe4* were all significantly lower than that of the T6SS-2-inactivated strain, ΔT6SS-2 (Figure 6A), indicating that mutants Δ*txe1* and Δ*txe4* possessed higher ability of bacterial killing than mutant ΔT6SS-2. Given that T6SS-2 is the sole T6SS present in *P. plecoglossicida* strain XSDHY-P involved in bacterial competition under the same experimental condition (Tao et al., 2020), these data suggest that the deletion of *txe1* or *txe4* alone did not completely abolish T6SS-2 killing.

Next, we tested whether *txe1* and *txe4* work synergically to mediate the observed bacterial killing in strain XSDHY-P. To this end, we created three multiple-effector deletion mutants including Δ*txe1*Δ*txe4* and Δ*txe1*Δ*txe2*Δ*txe4*. Then, we compared the PGI values of these mutants with that of ΔT6SS-2 in another interbacterial competition assay. The PGI of Δ*txe1*Δ*txe4* was not significantly different from those of ΔT6SS-2 or Δ*txe1*Δ*txe2*Δ*txe4* (Figure 6B), indicating that Δ*txe1*Δ*txe4* was attenuated as ΔT6SS-2, and Txe2 did not contribute to T6SS-2-mediated killing. Taken together, these results suggest that both Txe1 and Txe4 are used in the interbacterial competition by *P. plecoglossicida* strain XSDHY-P and that Txe1 is the predominant toxin responsible for killing *E. coli* in this assay.

## Discussion

Our previous work revealed that the fish pathogen, *P. plecoglossicida* strain XSDHY-P, encodes three T6SSs and T6SS-2 mediates interbacterial antagonism (Tao et al., 2020). In this work, we identified four putative antibacterial T6SS effectors that were named Txe1, Txe2, Txe3, and Txe4. The results presented here show that Txe1, Txe2, and Txe4 can effectively kill *E. coli* by exerting DNase activity when expressed in the cytosol (Figures 3A–C), suggesting that they are cytoplasmic-acting toxins. Although no conserved motif was identified in Txe1 CT domain based on the CDD search, a BLASTp search indicates that this nuclease might be commonly encoded with Rhs domain and in species belonging to the order Enterobacterales. Our site-directed mutagenesis analysis showed that one histidine residue (H1362) is essential for the toxicity of Txe1. However, more detailed analyses will be required to understand the exact role of His1362 in Txe1 and the corresponding residue in its homologs. Txe2 and Txe4 are AHH (pfam14412) and SHH (pfam15652) nuclease-domain containing effectors, respectively. Interbacterial competition assays show that *txe1* and *txe4* are necessary for the killing of *E. coli* in *P. plecoglossicida* strain XSDHY-P (Figures 6A,B). Although the data presented here largely confirm that Txe3 is not toxic to the *E. coli* strains used this study (Figure 5), we did not rule out the possibility that these particular *E. coli* strains have some T6SS-independent defense mechanisms that provide protection against activity of Txe3.

Txe1-Txi1, Txe2-Txi2, and Txe4-Txi4 are functional effector–immunity pairs. Our bioinformatic analysis revealed that these three effectors all contain a highly conserved Rhs core domain, but only Txe1 and Txe4 have a canonical Rhs-effector domain organization including a PAAR motif at the N-terminus, a domain of conserved Rhs-repeats in the central region, and a toxic domain at the C-terminus (Figures 3, 4). In addition, a conserved prePAAR motif and two transmembrane helices were identified in the region upstream of the respective PAAR domains of Txe1 and Txe4, a pattern that has also seen in other T6SS effectors. The prePAAR motif has been proposed to mediate effector–VgrG spike interactions (Ahmad et al., 2020), while the TMDs could facilitate the toxin entering into the inner membranes of target cells (Quentin et al., 2018). In contrast, Txe2 lacks the prePAAR motif and PAAR domain at its N terminus. It is still unclear whether the lack of PAAR domain influences the delivery of Txe2 in *P. plecoglossicida* and thereby restricts its role in the interbacterial competition, since PAAR is required for T6SS assembly in some species (Zheng et al., 2021). Our results regarding the function of these three toxins show that Txe1, Txe2, and Txe4 exhibit DNase activity and inhibit *E. coli* growth. Txe2 appears to be more toxic than Txe1 and Txe4, possibly due to differences in their mechanisms. In this study, the predicted immunity genes (*txi1*, *txi2*, and *txi4*) were co-expressed with cognate toxic effector genes in *E. coli* to prove that they are *bona fide* effector–immunity pairs. The putative immunity genes, when co-expressed, did not completely suppress plasmid DNA degradation by their cognate effectors in the cytoplasm; however, the toxicity of Txe1, Txe2, and Txe4 can be strikingly nullified based on growth curves and viable cell counts of *E. coli* strains (Figures 5A–F), confirming that Txe1-Txi1, Txe2-Txi2, and Txe4-Txi4 are functional effector–immunity pairs.

Txe1 is genetically linked to the *vgrG-2* cluster (encoded in the main T6SS-2 structural module), and Txe4 is encoded within *vgrG-5* cluster, which is not genetically linked to main T6SS-2 cluster. Two *vgrG* clusters have identical organization (one *vgrG*, two *eagR* genes, and downstream Rhs-toxin gene). In addition, VgrG, EagR, and Rhs effector counterparts encoded between the two gene clusters are also highly homologous (Supplementary Figures S1–S3), indicating that the two *vgrG* clusters are likely the result of a gene duplication event or are acquired from close relative by gene transfer. Together, these findings suggest that the proteins encoded in the *vgrG-2* and *vgrG-5* clusters may form VgrG-EagR-Rhs complexes of sufficient structural similarity to interact with the tube and baseplate of the same T6SS system (namely, T6SS-2) for toxin delivery (Jurėnas et al., 2021).

Txe1, Txe2, and Txe4 were identified to be homologous to Tke2 of *P. putida* strain KT2240 based on the analysis provided by the SecreT6 webtool (Li et al., 2015). Sequence alignment of the four effectors revealed that the sequence homology between Tke2 and Txe1, Txe2 or Txe4 was only found in the Rhs domains and not in the CT domains (Figure 2D). Tke2 is an effector deployed by K1-T6SS in *P. putida* KT2440 (Bernal et al., 2017). We analyzed the protein sequences of core structural components of T6SS-2 and K1-T6SS and found that they shared >90% amino acid sequence identity (Supplementary Table S4). We postulate that maintaining a conservative antibacterial T6SS across evolution confers a fitness advantage to these two *Pseudomonas* species, since the T6SS is energetically costly (Kudryashev et al., 2015). Nonetheless, *P. putida* and *P. plecoglossicida* have evolved their own distinctive toxin effectors and cognate immunity genes, and this may cause social incompatibilities and in turn lead to further genetic isolation (Vassallo et al., 2020).

## Conclusion

In summary, we identified three type VI secretion DNase toxin and immunity pairs (Txe1-Txi1, Txe2-Txi2 and Txe4-Txi4) in *P. plecoglossicida* strain XSDHY-P, and they are encoded in two *vgrG* clusters. Txe1, Txe2, or Txe4 expressed in the cytoplasm of *E. coli* displayed comparable toxicity against host *E. coli* cells, while only Txe1 and Txe4 were shown to mediate the interspecies antagonism of strain XSDHY-P during the competition with an *E. coli* strain. The findings of this study contribute to our understanding of the bacterial effectors involved in competition between bacteria.

## Data Availability Statement

The original contributions presented in the study are included in the article/Supplementary Material; further inquiries can be directed to the corresponding author.

## Author Contributions

ZT and XY conceived and designed the experiments. YL performed the experiments. ZT and YL analyzed the data and wrote the manuscript. All authors contributed to the article and approved the submitted version.

## Funding

This work was supported by Zhejiang Key Science and Technology Project (grant no. 2020C02004) and also supported by the Fundamental Research Funds for Zhejiang Provincial Universities and Research Institutes (grant no. 2021J012). The funders had no role in study design, data collection and analysis, decision to publish, or preparation of the manuscript.

## Conflict of Interest

The authors declare that the research was conducted in the absence of any commercial or financial relationships that could be construed as a potential conflict of interest.

## Publisher’s Note

All claims expressed in this article are solely those of the authors and do not necessarily represent those of their affiliated organizations, or those of the publisher, the editors and the reviewers. Any product that may be evaluated in this article, or claim that may be made by its manufacturer, is not guaranteed or endorsed by the publisher.
