# Supplementary material for: Two Type VI Secretion DNase Effectors are Utilized for Interbacterial Competition in the Fish Pathogen Pseudomonas plecoglossicida
Source: Front Microbiol. 2022 Apr 6;13:869278. doi: 10.3389/fmicb.2022.869278 (PMC9020831; doi:10.3389/fmicb.2022.869278)
Supplement: Supplementary file 1 [file Data_Sheet_1.docx]

Supplementary Material

# Supplementary Tables

**Supplementary Table S1**: Bacterial strains and plasmids used in this work.

| **Bacterial strain** | **Description^a^** | **Reference or source** |
| --- | --- | --- |
| *P. plecoglossicida* | | |
| XSDHY-P | Wild-type, fish pathogenic strain; Cam^R^ | (Zhang et al., 2014) |
| Δ*T6SS-2* | Deletion of whole T6SS-2 gene cluster | This work |
| Δ*txe1* | Deletion of *vgrG-2* associated effector | This work |
| Δ*txe2* | Deletion of *vgrG-2* associated effector | This work |
| Δ*txe3* | Deletion of *vgrG-4* associated effector | This work |
| Δ*txe4* | Deletion of *vgrG-5* associated effector | This work |
| Δ*txe1*Δ*txe2* | Deletion of *vgrG-2* associated effectors | This work |
| Δ*txe1*Δ*txe4* | Deletion of *vgrG-2* and *vgrG-5* associated effector | This work |
| Δ*txe1*Δ*txe2*Δ*txe4* | Deletion of *vgrG-2* and *vgrG-5* associated effectors | This work |
| *E. coli* | | |
| S17λπ | Host for conjugation | Laboratory collection |
| XL10 | prey strain for interbacterial competition assay | Vazyme |
| BL21(DE3) | Host of plasmid for expression | CWbio |
| **Plasmid** |  |  |
| pK18mob*sacB* | suicide plasmid containing Km^R^ and *sacB* gene for generating in frame deletion mutant | (Schäfer et al., 1994) |
| pK18-*Δtxe1* | For generating in frame deletion mutant of *txe1* | This work |
| pK18-Δ*txe2* | For generating in frame deletion mutant of *txe2* | This work |
| pK18-Δ*txe3* | For generating in frame deletion mutant of *txe3* | This work |
| pK18-Δ*txe4* | For generating in frame deletion mutant of *txe4* | This work |
| pBAD33.1 | Cam^R^, arabinose-inducible gene expression vector | (Chung and Raetz, 2010) |
| pTxe1 | Cam^R^, arabinose-inducible *txe1* expression vector | This work |
| pTxe2 | Cam^R^, arabinose-inducible *txe2* expression vector | This work |
| pTxe3 | Cam^R^, arabinose-inducible *txe3* expression vector | This work |
| pTxe4 | Cam^R^, arabinose-inducible *txe4* gene expression vector | This work |
| pTxe1^CT^ | Cam^R^, arabinose-inducible CT domain of *txe1* expression vector | This work |
| pTxe2^CT^ | Cam^R^, arabinose-inducible CT domain of *txe2* expression vector | This work |
| pTxe4^CT^ | Cam^R^, arabinose-inducible CT domain of *txe4* expression vector | This work |
| pTxe1^CT(H1362A)^ | Cam^R^, arabinose-inducible mutated *txe1* expression vector | This work |
| pTxe1-Txi1 | Cam^R^, arabinose-inducible *txe1-txi1* co-expression vector | This work |
| pTxe2-Txi2 | Cam^R^, arabinose-inducible *txe2-txi2* co-expression vector | This work |
| pTxe4-Txi4 | Cam^R^, arabinose-inducible *txe4-txi4* co-expression vector | This work |

CT: C-terminal domain; Cam^R^：chloramphenicol resistant；Km^R^：kanamycin resistant；Tc^R^：tetracycline resistant；

**References**

Chung, H.S., and Raetz, C.R. (2010). Interchangeable domains in the Kdo transferases of *Escherichia coli* and *Haemophilus influenzae*. *Biochemistry* 49**,** 4126-4137.

Schäfer, A., Tauch, A., Jäger, W., Kalinowski, J., Thierbach, G., and Pühler, A. (1994). Small mobilizable multi-purpose cloning vectors derived from the *Escherichia coli* plasmids pK18 and pK19: selection of defined deletions in the chromosome of *Corynebacterium glutamicum*. *Gene* 145**,** 69-73.

Zhang, J.T., Zhou, S.M., An, S.W., Chen, L., and Wang, G.L. (2014). Visceral granulomas in farmed large yellow croaker, *Larimichthys crocea* (Richardson), caused by a bacterial pathogen, *Pseudomonas plecoglossicida*. *Journal of Fish Diseases* 37**,** 113-121.

**Supplementary Table S2:** Primers used in this study

| **Primer name** | **5'-3' sequence** | **Description** |
| --- | --- | --- |
| Construction for Txe expression plasmids | | |
| Txe1-F | CGGATCCCGGCAAGCCCCCAGTTTCATATGAT | To generate pTxe1 by Gibson assembly |
| Txe1-R | GCAAATGGGTTTCGAAGCGGCCAGGTT |  |
| V-Txe1-F | GGGCTTGCCGGGATCCGAATTCGAGCTCC |  |
| V-Txe1-F | GCCGCTTCGAAACCCATTTGCTGTC |  |
| Txe2-F | CAGCAAATGGGTTGCATCACAGATAACCTGAGGCGT | To generate pTxe2 by Gibson assembly |
| Txe2-R | AATTCGGATCCCGTCCAAAAATATCTTTCCAGTTAGAGCTTCTAGGG |  |
| V-Txe2-F | GATATTTTTGGACGGGATCCGAATTCGAGCTCC |  |
| V-Txe2-R | ATCTGTGATGCAACCCATTTGCTGTCCACCAG |  |
| Txe3-F | ATTCGGATCCCGTAGCGAACTTCCAAAACCAGGGTAAATCT | To generate pTxe3 by Gibson assembly |
| Txe3-R | GCAAATGGGTAGTGAGGCGGCGCG |  |
| V-Txe3-F | GAAGTTCGCTACGGGATCCGAATTCGAGCTCC |  |
| V-Txe3-R | CCGCCTCACTACCCATTTGCTGTCCACCAG |  |
| Txe4-F | AATTCGGATCCCGATTAAACGCTCCCAAAGCCTCATAG | To generate pTxe4 by Gibson assembly |
| Txe4-R | GCAAATGGGTTTCGAAGCGGCCAGGTT |  |
| V-Txe4-F | GGAGCGTTTAATCGGGATCCGAATTCGAGCTCC |  |
| V-Txe4-R | GCCGCTTCGAAACCCATTTGCTGTCCACCAG |  |
| Txe1CT-F | ACTGTCGACTGGGTGCCTTGGGAGAAAAAT (SalI) | To generate pTxe1^CT^ |
| Txe1CT-R | ACTGCCGGAAGGGCCGCAAGCCCCCAGTTTCATATG (BglI) |  |
| Txe2CT-F | CATGTCGACGTGGTGTTCAAAAGCACTGGGGA (SalI)) | To generate pTxe2^CT^ |
| Txe2CT-R | CATGCCGGAAGGGCCCAAAAATATCTTTCCAGTTAGAG (BglI) |  |
| Txe4CT-F | CATGTCGACGTGCTGGGCTTGTAATAAGCCTGG (SalI) | To generate pTxe4^CT^ |
| Txe4CT-R | ACTGCCGGAAGGGCTTAAACGCTCCCAAAGCCTCATA (BglI) |  |
| Site directed mutagenesis | | |
| Txe1CT-F | ACTGTCGACTGGGTGCCTTGGGAGAAAAAT (SalI) | To generate pTxe1^CT(H1362A)^ |
| Txe1M-US-R | CACTACTCCCATGGGCTGGACCATCAGGCACAG |  |
| Txe1M-DS-F | CTGTGCCTGATGGTCCAGCCCATGGGAGTAGTG |  |
| Txe1CT-R | ACTGCCGGAAGGGCCGCAAGCCCCCAGTTTCATATG (BglI) |  |
| Txe1M-DT-F | TCCTGTGCCTGATGGTCCAGCG | To detect pTxe1 ^CT(H1362A)^ |
| Txe1M-DT-R | TTAGCAAGCCCCCAGTTTCATA |  |
| Construction for Txe-Txi dual-expression plasmids | | |
| Txei1-F | TCGGATCCCGTCAATTCCCCTCAAAAAGCCCAATATATTTCAAG | To generate pTxe1-Txi1 by Gibson assembly |
| Txei1-R | GCAAATGGGTTTCGAAGCGGCCAGGTT |  |
| V-Txei1-F | GGGGAATTGACGGGATCCGAATTCGAGCTCC |  |
| V-Txei1-R | GCCGCTTCGAAACCCATTTGCTGTCCACCAG |  |
| Txei2-F | AGCAAATGGGTTGCATCACAGATAACCTGAGGCGT | To generate pTxe2-Txi2 by Gibson assembly |
| Txei2-R | TCGGATCCCGTTAAACCGCTCTGACTTCCTTGAAATCCA |  |
| V-Txei2-F | GCGGTTTAACGGGATCCGAATTCGAGCTCC |  |
| V-Txei2-R | ATCTGTGATGCAACCCATTTGCTGTCCACCAG |  |
| Txei4-F | AATTCGGATCCCGTTACTTAAACCGAATAATATGCGAATACTTTAGCAGC | To generate pTxe4-Txi4 by Gibson assembly |
| Txei4-R | GCAAATGGGTTTCGAAGCGGCCAGGTT |  |
| V-Txei4-F | CGGTTTAAGTAACGGGATCCGAATTCGAGCTCC |  |
| V-Txei4-R | GCCGCTTCGAAACCCATTTGCTGTCCACCAG |  |
| Generation of effector-deletion mutants | | |
| Txe1-US-F | ATAGGATCCTCAAGGGCAAGGTCATCGTCAC (BamHI) | To amplify upstream fragment of *txe1* |
| Txe1-US-R | ATATCCACTTAGCATTCGAACATGAGGGGTTACTCCTG |  |
| Txe1-DS-F | CCCCTCATGTTCGAATGCTAAGTGGATATTTATGATATCGAGAGGTAT | To amplify downstream fragment of *txe1* |
| Txe1-DS-R | GCATCTAGATCACTACCCGCTTGATGTCGG (XbaI) |  |
| Txe1-DT-F | CGTAGACGAAGTCCTGTCGG | To detect the Δ*txe1* mutant |
| Txe1-DT-R | CTTAACCAGACCACCACGCT |  |
| Txe2-US-F | TATCGAGAGGTATGAAAAAATT | To amplify upstream fragment of *txe2* |
| Txe2-US-R | ATAGTCATCCAAAGCACATGAACTCGCTGAATTAGAGAC |  |
| Txe2-DS-F | GCGAGTTCATGTGCTTTGGATGACTATGAAGTACTATTCTATAACCCAAGATAGT | To amplify downstream fragment of *txe2* |
| Txe2-DS-R | AGGCGACCCAGCGGATTGTCGTA |  |
| Txe2-V-F | GGGTCGCCTCCAGTCACGACGTTGTAAAACGAC | To generate Δ*txe2* mutation plasmid by Gibson assembly |
| Txe2-V-R | CATCAGGCACAGAAAACCCTGGCGTTACCCAAC |  |
| Txe2-P-F | ACATGATTACGAGGCGACCCAGCGG |  |
| Txe2-P-R | CCGAGCTCGAATTTATCGAGAGGTATGAAAAAATTCATGTGTGGG |  |
| Txe2-DT-F | AGGCGACATATCGCTCTTGG | To detect the Δ*txe2* mutant |
| Txe2-DT-R | GCTTTCAACACCGCGTCTTT |  |
| Txe3-US-F | CATGAATTCAACTTGAGCGTGAGCGTTTGG (EcoRI) | To amplify upstream fragment of *txe3* |
| Txe3-US-R | CCTTTCATAGCGACTCACTCATACCGGCGCG |  |
| Txe3-DS-F | CGGTATGAGTGAGTCGCTATGAAAGGTATCATTGAGGAAAATATTTTAAGGT | To amplify downstream fragment of *txe3* |
| Txe3-DS-R | CATGGATCCTGCCATCACCTGTTACAGCAAAAC (BamHI) |  |
| Txe3-DT-F | GCTCGAGCAATGTGTTCGTC | To detect the Δ*txe3* mutant |
| Txe3-DT-R | TTGACCCCTAGTCCTCCGTT |  |
| Txe4-US-F | GCAGAATTCGGTGCTGAGTCTATGGCTGGT (EcoRI) | To amplify upstream fragment of *txe4* |
| Txe4-US-R | AAATCACCATTAATTAAATTCGAACATGAGGGGTTACTCCTG |  |
| Txe4-DS-F | GTAACCCCTCATGTTCGAATTTAATTAATGGTGATTTATACTGAGCGTCGT | To amplify downstream fragment of *txe4* |
| Txe4-DS-R | CATGGATCCGCGAATACTTTAGCAGCGAATCCC (BamHI) |  |
| Txe4-DT-F | GAGTACCGCTATGACCCGTG | To detect the Δ*txe4* mutant |
| Txe4-DT-R | TGTTGTAGTGCAGCCGTGAT |  |
| Identification for *P. plecoglossicida* | | |
| PLG2-F | TGCTGAAGGACGAGCGTTCG | To target the *gyrB* gene of *P. plecoglossicida* strains for species identification. |
| PLG2-R | ATCATCTTGCCGACAACAGC |  |

V: vector; M: mutation; US: upstream ; DS: downstream ; DT: detection.

Restriction enzyme sites in primers are underlined.

**Supplementary Table S3:** Features encoded within the *P. plecoglossicida* XSDHY-P *vgrG* clusters

| **Gene**  **locus** | **Protein name** | **Conserved Domains  COG/pfam/TIGR  (Short Name)** | **Predicted**  **sub-cellular  localization^a, b^** | **Transmembrane  helices^c^** | **Predicted signal  peptide^d, e^** |
| --- | --- | --- | --- | --- | --- |
| ***vgrG-2* cluster** | | | | | |
| DVB73_RS_03045 | Txi2 | pfam09535  (Gmx_para_CXXCG) | 1. Cytoplasmic 2. Unknown | 0 | No No |
| DVB73_RS_03050 | Txe2 | COG3209 (RhsA)/ TIGR03696 (Rhs_assc_core) + pfam14412 (AHH) | 1. Outer membrane 2. Cytoplasmic | 0 | No No |
| DVB73_RS_03055 | Txi1 | pfam14112 （DFU4284) | 1. Cytoplasmic 2. Unknown | 0 | No No |
| DVB73_RS_03060 | Txe1 | pfam05488 (PAAR_motif) + COG3209 (RhsA)/TIGR03696 (Rhs_assc_core) | 1. Inner membrane 2. Unknown | 2 | No No |
| DVB73_RS_03065 | EagR-2b | pfam08786 (DcrB) | 1. Cytoplasmic 2. Unknown | 0 | No No |
| DVB73_RS_03070 | EagR-2a | - | 1. Cytoplasmic 2. Cytoplasmic | 0 | No No |
| DVB73_RS_03075 | VgrG-2 | COG3501 (VgrG)/ pfam05954 (Phage_GPD)/ TIGR03361 (VI_Rhs_Vgr) + pfam10106 (DUF2345) | 1. Extracellular 2. Cytoplasmic | 0 | No No |
| DVB73_RS_03080 |  | - | 1. Inner membrane 2. Outer membrane | 5 | No No |
| DVB73_RS_03085 |  | - | 1. Inner membrane 2. Unknown | 1 | No No |
| ***vgrG-4* cluster** | | | | | |
| DVB73_RS_04040 | Txi3 | - | 1. Cytoplasmic 2. Cytoplasmic | 0 | No No |
| DVB73_RS_04045 | Txe3 | COG4104 (PAAR)/ pfam05488 (PAAR_motif)+ pfam01844 (HNHc) | 1. Cytoplasmic 2. Cytoplasmic | 0 | No No |
| DVB73_RS_04050 | EagR | COG5435/ pfam08786 (DcrB) | 1. Inner membrane 2. Outer membrane | 3 | No No |
| DVB73_RS_04055 | VgrG-4 | COG3501 (VgrG)/ pfam05954 (Phage_GPD)/ TIGR03361 (VI_Rhs_Vgr) | 1. Cytoplasmic 2. Unknown | 0 | No No |
| ***vgrG-5* cluster** | | | | | |
| DVB73_RS_08325 | Txi4 | pfam09346 (SMI1_KNR4) | 1. Cytoplasmic 2. Cytoplasmic | 0 | No No |
| DVB73_RS_08330 | Txe4 | COG4104 (PAAR)/ pfam05488 (PAAR_motif)+ COG3209 (RHS)/ TIGR03696 (Rhs_assc_core)  pfam15652 (Tox-SHH) | 1. Inner membrane 2. Outer membrane | 2 | No No |
| DVB73_RS_08335 | EagR-5b | COG5435 (COG5435)/ pfam08786 (DcrB) | 1. Cytoplasmic 2. Unknown | 0 | No No |
| DVB73_RS_08340 | EagR-5a | - | 1. Cytoplasmic 2. Cytoplasmic | 0 | No No |
| DVB73_RS_08345 | VgrG-5 | COG3501 (VgrG)/ pfam05954 (Phage_GPD)/ TIGR03361 (VI_Rhs_Vgr) + pfam10106 (DUF2345) | 1. Extracellular 2. Cytoplasmic | 0 | No No |

a: The PSORTb server (<https://www.psort.org/psortb/index.html>) was used to predict the subcellular localizations.

b: The SOSUIGramN tool (<https://harrier.nagahama-i-bio.ac.jp/sosui/sosuigramn/sosuigramn_submit.html>) was used to predict the subcellular localizations.

c: The TMHMM 2.0 (https://services.healthtech.dtu.dk/service.php?TMHMM-2.0) was used to predict the presence of transmembrane domains.

d: The SignalP 5.0 server (https://services.healthtech.dtu.dk/service.php?SignalP-5.0) was used to predict the presence of signal peptides.

e: The SOSUIsignal (<http://bp.nuap.nagoya-u.ac.jp/sosui/sosuisignal/sosuisignal_submit.html>) was used to predict the presence of signal peptide

**Table S4:** Features encoded within the *P. plecoglossicida* XSDHY-P T6SS-2 cluster

| **Locus name** | **Protein name** | **Protein length** | **Identities with**  ***P. putida* KT2440**  **K1-T6SS  %BLASTp identity/%coverage** | **Conserved Domains** |
| --- | --- | --- | --- | --- |
| DVB73_RS03175 | TssA-2 | 365 | PP3088 (TssA1)  82/98 | COG3515/pfam06812 (ImpA-rel_N) + TIGR03363 (VI_chp_8) |
| DVB73_RS03170 | TssD-2/Hcp-2 | 172 | PP3089 (TssD1) 97/100 | COG3157 (Hcp)/ pfam05638 (DUF796)/ TIGR03344 (VI_effect_Hcp1) |
| DVB73_RS03165 | TagP-2 | 831 | PP3090 (TagP1) 32/43 | COG3523 (IcmF)/ pfam14331 (ImcF-related_N)/TIGR03348 (VI_IcmF) + pfam00691 (OmpA) |
| DVB73_RS03160 | TagF-2 | 302 | PP3090.1 (TagF1) 81/100 | pfam (DUF2094) |
| DVB73_RS03155 | TssM-2 | 1267 | PP3091 (TssM1) 89/100 | COG3523 (IcmF)/ pfam14331 (ImcF-related_N)/TIGR03348 (VI_IcmF) |
| DVB73_RS03150 | TssL-2 | 238 | PP3092 (TssL1) 92/100 | COG3455/pfam09850 (DUF2077)/ TIGR03349 (TIGR03349) |
| DVB73_RS03145 | TssK-2 | 447 | PP3093 (TssK1) 95/100 | COG3522/ pfam05936 (DUF876)/TIGR03353 (VI_chp_4) |
| DVB73_RS03140 | TssJ-2 | 243 | PP3094 (TssJ1) 80/100 | COG3521/pfam12790 (T6SS-SciN)/TIGR03352 (VI_chp_3) |
| DVB73_RS03135 | TssH-2/ClpV-2 | 865 | PP3095 (TssH1) 93/100 | COG0542 (ClpA) + pfam07724 (AAA_2)/ TIGR03345 (VI_ClpV1) |
| DVB73_RS03130 | TssG-2 | 356 | PP3096 (TssG1) 94/100 | COG3520/pfam06996 (DUF1305)/TIGR03347 (VI_chp_1) |
| DVB73_RS03125 | TssF-2 | 606 | PP3097 (TssF1) 94/100 | COG3519/pfam05947 (DUF879)/TIGR03359 (VI_chp_6) |
| DVB73_RS03120 | TssE-2 | 160 | PP3098 (TssE1) 96/100 | COG3518/ pfam04965 (GPW_gp25)/ TIGR03357 (VI_zyme) |
| DVB73_RS03115 | TssC-2 | 500 | PP3099 (TssC1) 97/100 | COG3517/pfam05943 (DUF877)/TIGR03355 (VI_chp_2) |
| DVB73_RS03110 | TssB-2 | 177 | PP3100 (TssB1) 95/99 | COG3516/pfam05591 (DUF770)/TIGR03358 (VI_chp_5) |
| DVB73_RS03105 | TagF-2 | 249 | PP3100.1 (TagX1) 90/100 | -- |

# Supplementary Figures


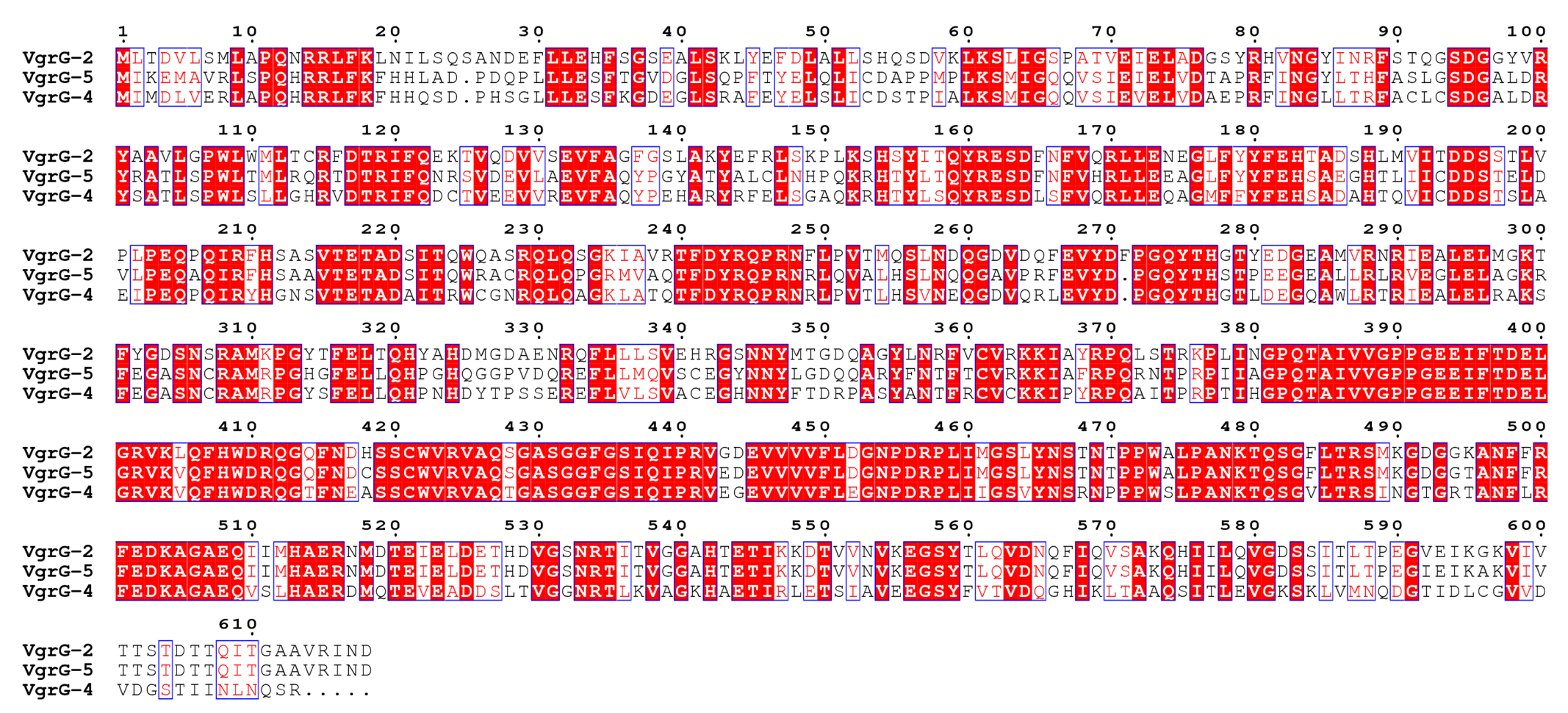


**Supplementary Figure S1.** Amino acid sequence alignments of VgrG-2, VgrG-4, and VgrG-5 proteins. Conserved and similar residues are shown in red and blue boxes, respectively. Strictly conserved (white text with red background) and 75% conserved or similarly substituted (red text with white background) residues are shown. The sequence alignment was visualized with ESPript using the new ENDscript server (Robert and Gouet, 2014).


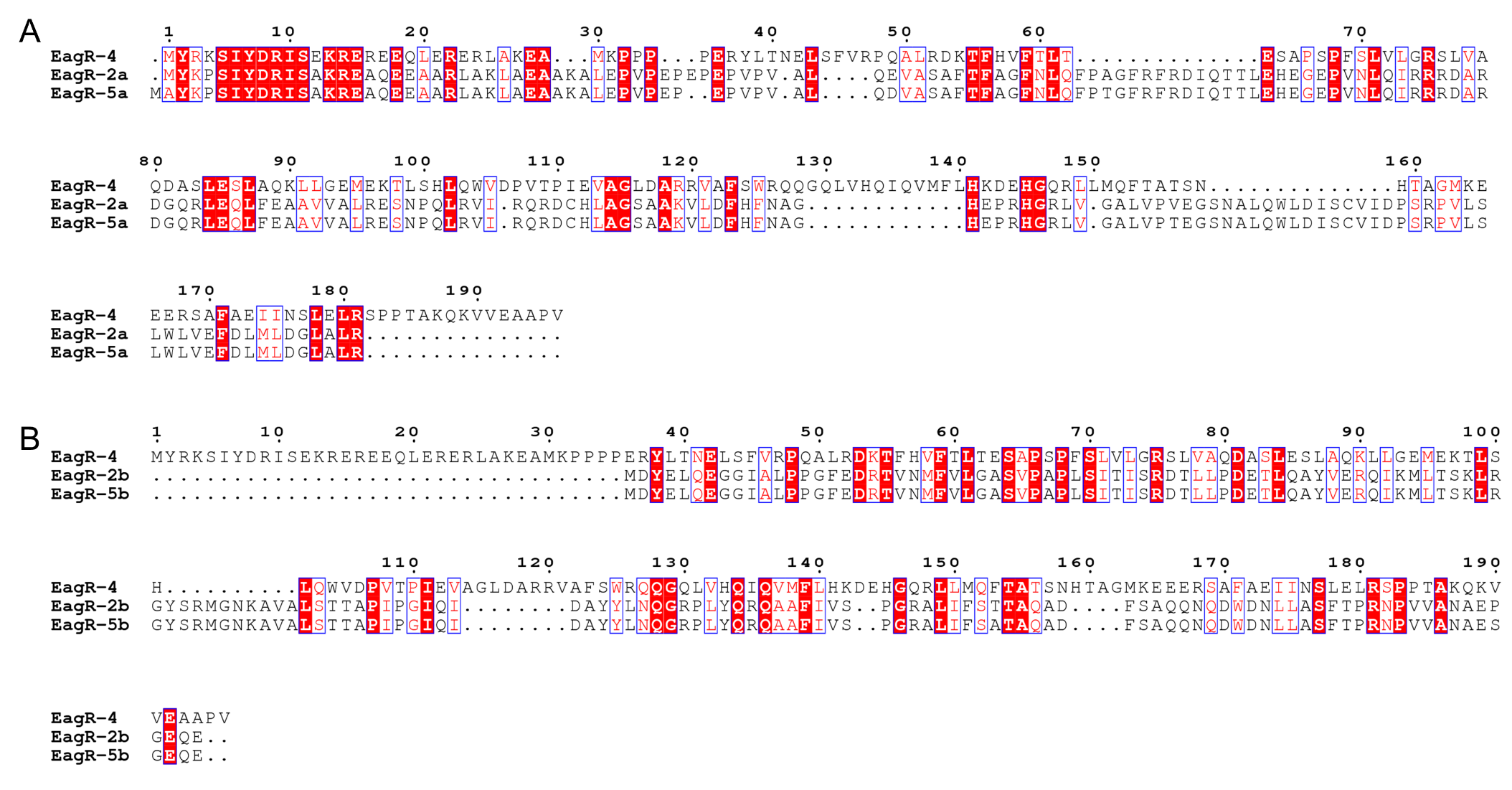


**Supplementary Figure S2.** Amino acid sequence alignment of EagR chaperones encoded in three identified *vgrG* clusters. Sequence alignment of EagR-4, EgaR-2a and EgaR-5a **(A)**. Sequence alignment of EagR-4, EagR-2b and EagR-5b **(B).** Conserved and similar residues are shown in red and blue boxes, respectively. Strictly conserved (white text with red background) and 75% conserved or similarly substituted (red text with white background) residues are shown. The sequence alignment was visualized with ESPript using the new ENDscript server (Robert and Gouet, 2014).


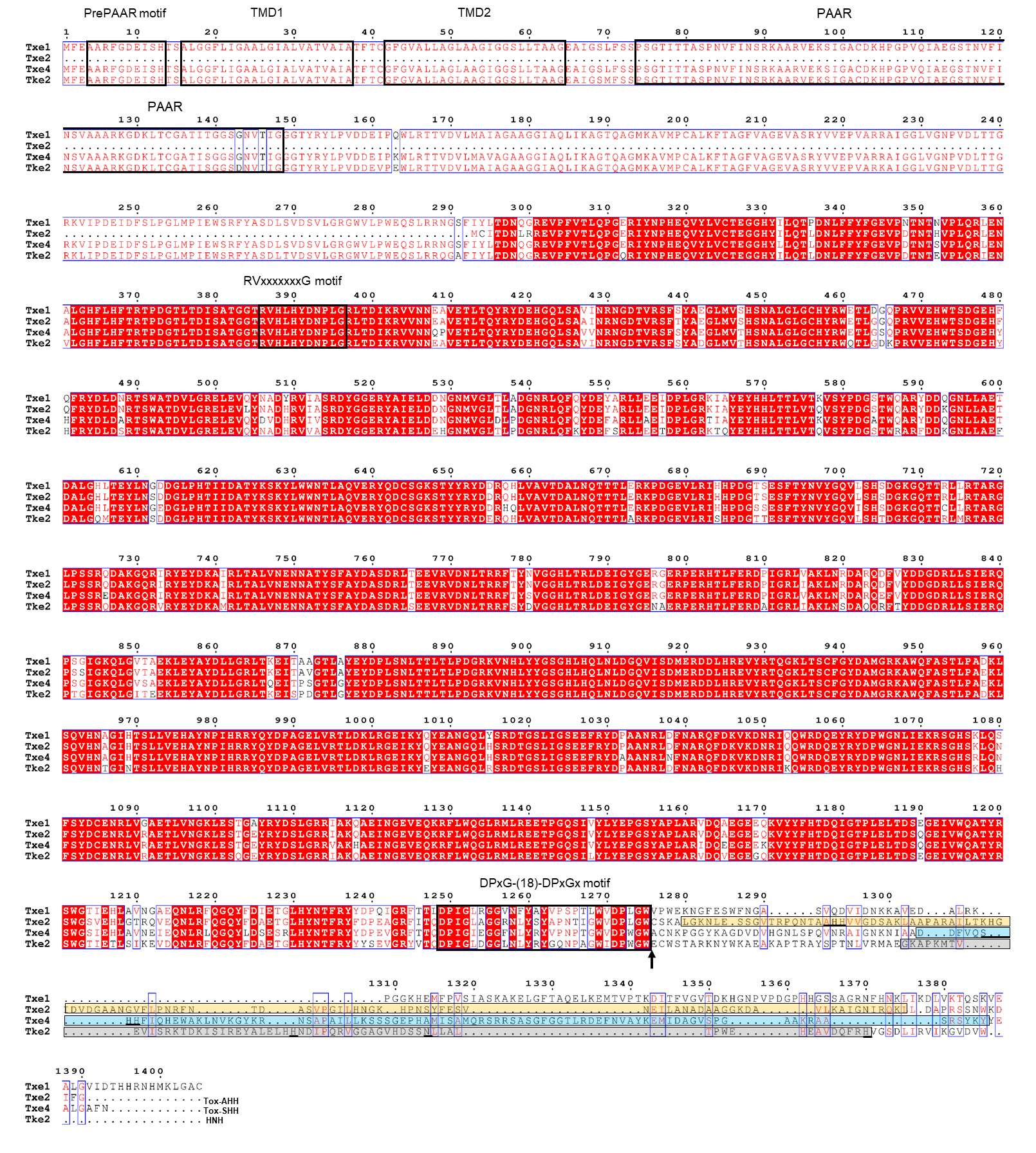
**Figure S3.** Amino acid sequence alignment of putative effectors Txe1, Txe2 and Txe4 of *P. plecoglossicida* XSDHY-P and Tke2 from *P. putida* KT2440 (Bernal et al., 2017). Numbers refer to the Txe1 sequence. The RV*xxxxxxxx*G and DPxG-(18)-DPxGx motifs that demarcate the Rhs core domains are indicated with a line on the top. The black arrow indicates the predicted cleavage site between the C-terminal domain and Rhs core domain (Pei et al., 2020). Conserved and similar residues are shown in red and blue boxes, respectively. Strictly conserved (white text with red background) and 75% conserved or similarly substituted (red text with white background) residues are shown. The

The sequence alignment was visualized with ESPript using the new ENDscript server (Robert and Gouet, 2014).


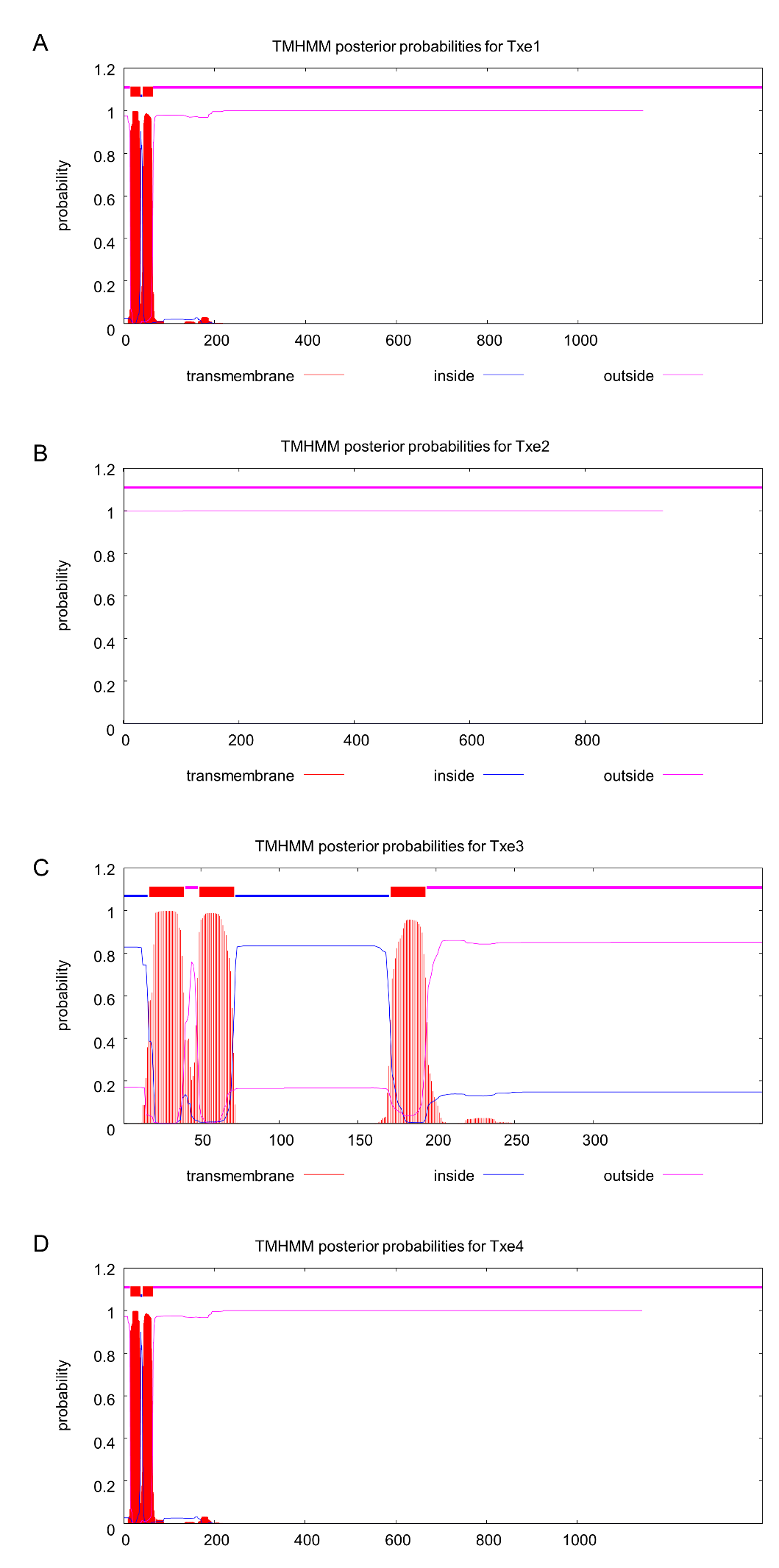


**Supplementary Figure S4.** Prediction of transmembrane helices in Txe1, Txe2, Txe3 and Txe4 **(A-D)**. Red regions indicate putative transmembrane domains with the relative probability of each indicated on the Y-axis. Pink and blue regions correspond to predicted extracellular and intracellular segments respectively. TMHMM Server v.2.0 (http://www.cbs.dtu.dk/services/TMHMM) was used for predictions.


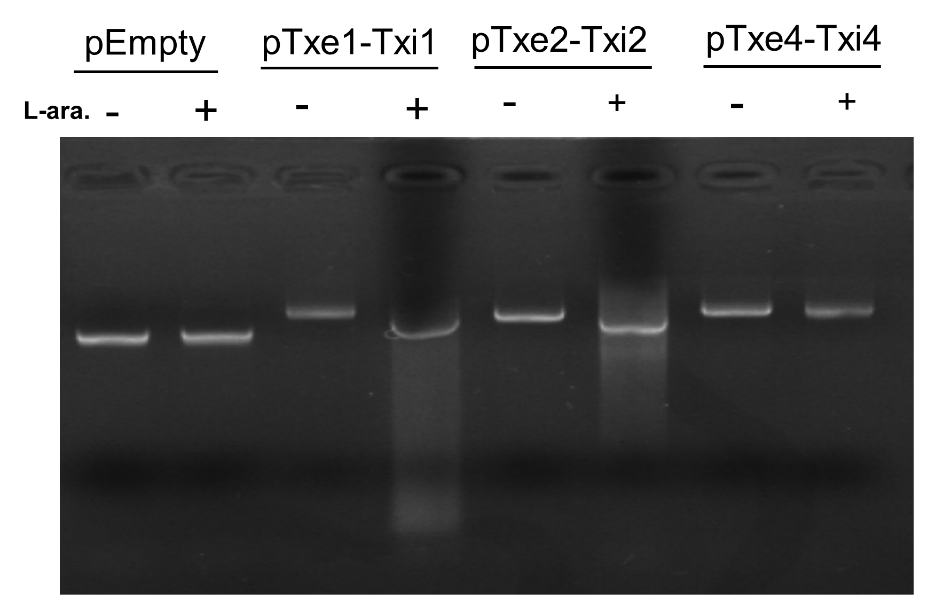


**Supplementary Figure S5.** Nuclease activity assay. *E. coli* cells with pBAD33.1 plasmid (pEmpty) and effector with immunity protein (Txe1-Txi1, Txe2-Txe2 or Txe4-Txi4) were induced with (+) or without (–) 0.2% L-arabinose (L-ara) for 3 h. Plasmid DNA was extracted and the degradation pattern was observed in agarose gel.
